# Study on the Properties of High Fly Ash Content Alkali-Activated Fly Ash Slag Pastes and Fiber-Reinforced Mortar Under Normal Temperature Curing

**DOI:** 10.3390/ma17225668

**Published:** 2024-11-20

**Authors:** Zhu Yuan, Yanmin Jia, Jinyu Sun, Xiaobo Zhang, Yaojie Hu, Xuhua Han

**Affiliations:** 1 School of Civil Engineering and Transportation, Northeast Forestry University, Harbin 150040, China; zhu_yuan163@163.com; 2 Qingdao Transportation Bureau, Qingdao 266061, China; 3 Jiaozhou Highway Development Center, Qingdao 266399, China; 4 Transportation Bureau of Chengyang District, Qingdao 266107, China; 5 Qingdao Transportation Planning and Design Institute Co., Ltd., Qingdao 266075, China

**Keywords:** alkali-activated fly ash slag composite system, normal temperature curing, high content fly ash, glass fiber, polypropylene fiber

## Abstract

In order to efficiently utilize industrial solid waste while minimizing the preparation cost of engineering materials and the technical difficulty of construction, this paper prepared a high fly ash content alkali-activated fly ash slag composite system at normal temperatures and conducted an in-depth investigation on it. A systematic study was conducted on the workability, mechanical properties, and microstructures of the alkali-activated fly ash slag pastes, including setting times, strength, phase, and molecular structures. We then designed and prepared fiber-reinforced alkali-activated fly ash slag mortar and studied the effects of the alkali activator modulus, glass fiber (GF), and polypropylene fiber (PPF) on the workability, mechanical properties, and frost resistance of the mortar. The following main conclusions were drawn: By adjusting the modulus of alkali activator for alkali-activated fly ash slag pastes, characteristics that meet engineering requirements could be obtained. The compressive strength of the pastes decreased with increasing proportions of fly ash, and it first increased and then decreased with increases in the activator modulus. The flexural strength decreased to varying degrees as the modulus of the activator increased. Through SEM, fly ash particles with different reaction degrees could be observed, indicating that the reaction was still ongoing. The addition of GF and PPF reduced the fluidity of mortar and significantly improved its strength and frost resistance. Fiber had the most significant effect on improving the strength of the mortar, as an activator modulus of 1.0. 0.45% PPF increased the flexural and compressive strength of the mortar by 14.33% and 29.1%, respectively, while 0.90% GF increased the flexural and compressive strength of the mortar by 3.12% and 19.21%, respectively. The frost resistance of the mortar with an activator modulus of 1.0 was significantly better than that of the mortar with an activator modulus of 1.4. 0.45% PPF and reduced the quality loss rate of the mortar by 49.30%, effectively delaying the deterioration of its freeze-thaw performance.

## 1. Introduction

With the development of transportation infrastructure construction in China, the demand for cement concrete continues to increase, resulting in a high level of production and consumption of cement. According to statistical data, national cement production reached 2.023 billion tons in 2023. The production of cement is accompanied by high-energy consumption and significant greenhouse gas emissions. For every ton of cement produced, approximately 0.8 tons of CO_2_ are emitted into the atmosphere [1,2], and nearly 8% of global CO_2_ emissions are generated by the cement industry [3]. With the increasing awareness of environmental protection and sustainable development, it is crucial to develop new cementitious materials to replace cement in order to reduce its carbon footprint.

Alkali-activated materials (AAMs) refer to a cementitious system formed by the reaction of alkali metal sources (solid or solution) with solid silicate powder materials [2].

Compared with ordinary Portland cement concrete, AAMs have been considered as a cement substitute that can significantly reduce CO_2_ emissions in the preparation of concrete [4,5,6,7,8,9,10,11], with properties that are “completely equivalent to excellent Portland cement” [12].

Fly ash and granulated blast furnace slag, as industrial solid waste, currently have high emissions but relatively low efficient utilization rates. The accumulation of industrial solid waste occupies a large amount of arable land and other land resources, and dust causes serious environmental pollution [13,14,15]. Using alkaline activators to stimulate industrial waste and develop alternatives to cement is one of the strategies with broad prospects. It can not only reduce CO_2_ emissions but also helps alleviate environmental and economic pressures caused by the disposal of industrial solid waste.

Fly ash and granulated blast furnace slag contain abundant volcanic ash components such as calcium oxide, silicon dioxide, and aluminum oxide. Fly ash also contains a large amount of amorphous silicon aluminum substances, making it a precursor for AAM. Slag has high reactivity and can form a large amount of cementitious substances under the action of a small amount of activators (such as Ca (OH)_2_, NaOH), thereby exhibiting considerable hydraulic hardness. It is suitable as an active mixing material in cement or a mineral admixture in concrete. The finer the slag, the higher its activity index, but the corresponding hydration heat and shrinkage also increase.

Based on previous research, AAMs using slag as a precursor have poor workability, short setting times, and large amounts of shrinkage [16,17,18,19,20], while AAMs using fly ash as a precursor have slow strength development at room temperature. Due to the high excitation energy of fly ash, alkali-activated cementitious materials based on fly ash require high amounts of activators and high-temperature curing to achieve satisfactory mechanical properties such as early strength [21,22,23,24,25,26,27,28]. Some researchers have also attempted to use direct current [29,30,31,32] or microwave curing [33,34,35,36,37,38] to accelerate the reaction process of alkali-activated fly ash composite systems.

However, compared with ambient temperature curing, curing methods such as high-temperature curing, direct current curing, or microwave curing require more energy and increase carbon dioxide emissions. In addition, the curing process will increase construction costs and technical difficulties, which may reduce the applicability of AAMs in the field of civil engineering. Therefore, normal temperature curing is crucial for the various commercial uses and promotion of AAMs [5,39].

Fly ash and slag have a good synergistic effect in alkali-activated reactions [7,40,41,42,43], which can achieve good mechanical strength and durability. Slag/fly ash-based alkali-activated cementitious materials have the advantages of fast strength gain, fire resistance, weathering resistance, salt resistance, and acid resistance [44,45,46,47,48,49,50,51,52,53], and have therefore received widespread attention from researchers.

Although alkali-activated fly ash slag cementitious materials have shown broad application prospects, in most current research, the proportion of fly ash in the precursor is relatively low. In China, the production and stocking of fly ash far exceed that of slag. However, the efficient utilization rate of fly ash in building materials is far lower than the latter, which is not conducive to the comprehensive utilization of fly ash. 

Therefore, by increasing the dosage of fly ash, high fly ash content alkali-activated fly ash slag composite materials can be prepared. At present, there is still a lack of systematic research on the workability, mechanics, and durability of such materials.

Based on the efficient utilization of industrial solid waste and the reduction of engineering material preparation costs and construction technology difficulties, this paper designed and prepared an alkali-activated fly ash slag composite system. The fly ash content was significantly increased from 75% to 85% of the precursor dosage. Under the premise of normal temperature curing, alkali-activated fly ash slag pastes and fiber-reinforced mortar were prepared. A systematic study was conducted on the workability, mechanical properties, and microstructures of the pastes, including setting times, flexural strength, and compressive strength. The phase, molecular structure, and microstructure of the pastes were analyzed using X-ray diffraction (XRD), Fourier transform micro infrared spectroscopy (FT-IR), and scanning electron microscopy (SEM). On this basis, the workability, mechanical properties, and frost resistance of fiber-reinforced alkali-activated fly ash slag mortar were studied. This study provides a research foundation for the promotion and application of this type of green building material in civil engineering construction.

## 2. Raw Materials and Mix Proportions

### 2.1. Raw Materials

The selected fly ash came from the Harbin Shuangda Fly Ash Products Factory and belongs to the first-grade low-calcium fly ash class (with a total content of Al_2_O_3_, SiO_2_, and Fe_2_O_3_ exceeding 70%), with a density of 2.30 g/cm^3^ (The density information comes from the manufacturer). The slag used was an S95-grade slag produced by Hebei Jinghang Mineral Products Co., Ltd., with a density of 2.90 g/cm^3^ (The density information comes from the manufacturer) and a specific surface area of 418 m^2^/kg. The chemical composition of fly ash and slag was detected using a ZSXPrimus II X-ray fluorescence spectrometer (Rigaku Corporation, Tokyo, Japan), and the results are shown in Table 1.

**Figure 1:**
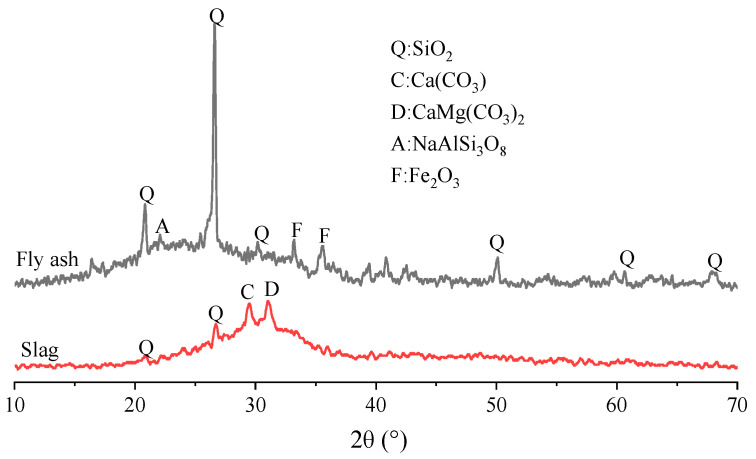
XRD analysis of raw materials fly ash and slag.

Figure 1 shows the XRD test results of raw materials fly ash and slag. Fly ash mainly contained amorphous substances and components such as quartz (SiO_2_), sodium feldspar (NaAlSi_3_O_8_), hematite (Fe_2_O_3_), etc. The slag mainly contained quartz (SiO_2_), calcite (CaCO_3_), dolomite (CaMg (CO_3_)_2_), and other components.

**Figure 2:**
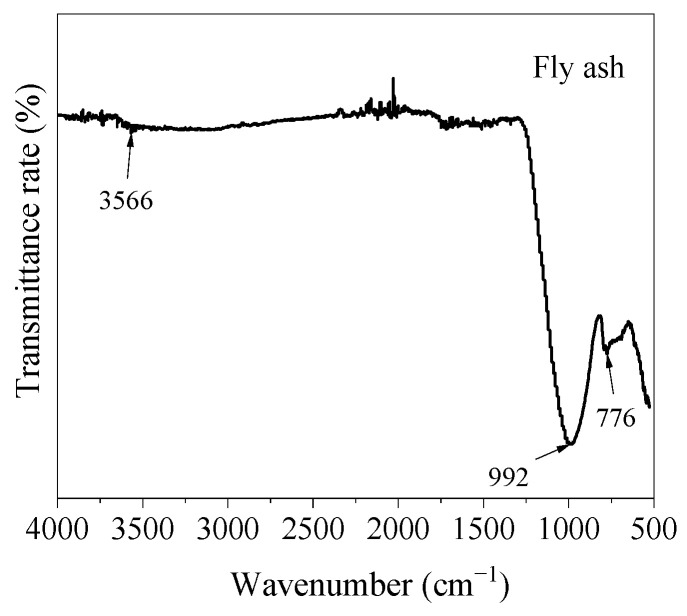
FT-IR analysis of raw material fly ash.

Figure 2 shows the FT-IR spectrum of the raw material fly ash. The absorption peak of fly ash at 776 cm^−1^ was the Si-O-Si bending vibration band, and there was a clear absorption peak at 992 cm^−1^, which belonged to Si-O stretching vibration. The absorption peak at 3566 cm^−1^ was the stretching vibration of non-hydrogen-bonded or weakly hydrogen-bonded hydroxyl groups in Ca-OH bonds.

**Figure 3:**
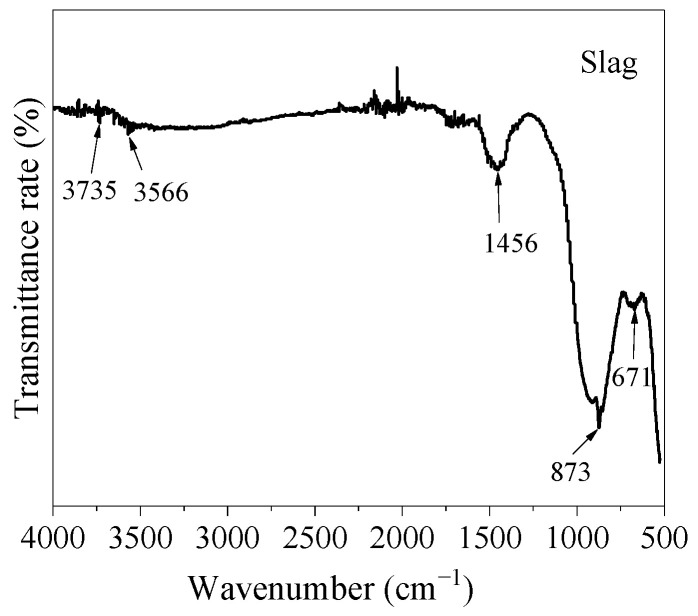
FT-IR analysis of raw material slag.

Figure 3 shows the FT-IR spectrum of the raw material slag. The absorption peak at 671 cm^−1^ was attributed to the Si-O-Si bending band, while the absorption peaks at 873 cm^−1^ and 1456 cm^−1^ corresponded to the asymmetric stretching vibration of O-C-O in carbonates. Similar to fly ash, the absorption peak at 3566 cm^−1^ was the stretching vibration of non-hydrogen-bonded or weakly hydrogen-bonded hydroxyl groups in Ca-OH bonds. The weak absorption peak at 3735 cm^−1^ was caused by the stretching of SiO-H in the SiOH group.

The selected alkaline activators were sodium silicate and sodium hydroxide, because these two alkaline activators are the most common, efficient, relatively inexpensive, easy to produce, and the combination of sodium hydroxide and sodium silicate can more effectively stimulate the strength of AAM. The addition of sodium hydroxide can change the SiO_2_/Na_2_O ratio of a sodium silicate solution, thereby creating favorable conditions for the alkaline activation reaction of the precursor.

The sodium silicate used was produced by the Tongxiang Hengli Chemical Co., Ltd. (implementing standard: GB/T 4209-2008 [54]), and its appearance was a slightly colored semi-transparent viscous liquid. The basic parameters of sodium silicate are shown in Table 2. Sodium hydroxide was produced by the Inner Mongolia Junzheng Energy Chemical Group Co., Ltd. (implementing standard: GB209-2018 [55]), with a purity of not less than 96%, and its appearance was a white opaque sheet-like solid.

**Figure 4:**
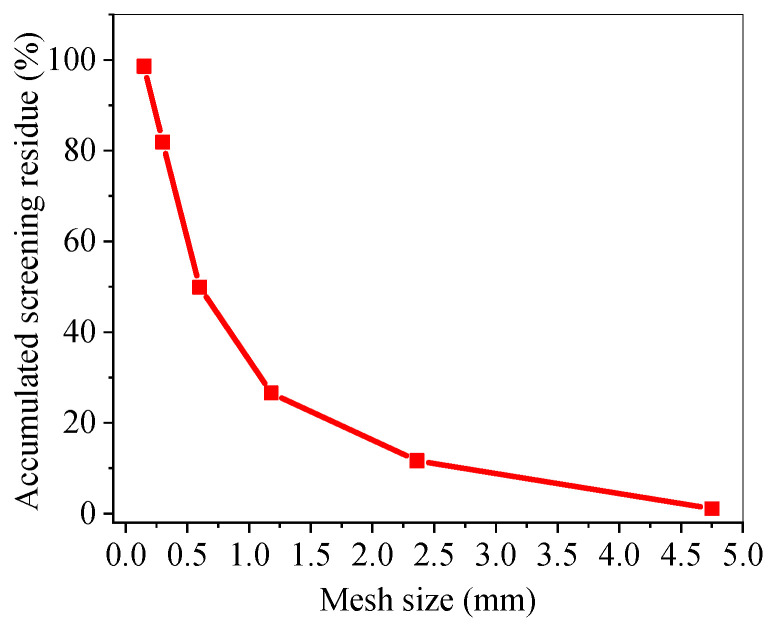
Grading curve of sand.

In the raw materials of mortar, natural river sand with a fineness modulus of 2.66 was selected as the fine aggregate, and the grading curve of sand is shown in Figure 4. The fibers used were short cut GF and PPF with a length of 12 mm. The appearance and main parameters of the fibers refer to the author’s previous research [56].

### 2.2. Mix Proportions

In alkali-activated fly ash slag pastes, the proportion of fly ash in the precursor (i.e. the sum of fly ash and slag) was set to 75%, 80%, and 85%. A mixed solution of sodium hydroxide and liquid sodium silicate was used as the alkaline activator. The modulus of the alkaline activator, i.e. the SiO_2_/Na_2_O ratio in the solution, was 0.6, 0.8, 1.0, 1.2, and 1.4. The mix proportions of alkali-activated fly ash slag pastes are shown in Table 3.

On the basis of the three sets of pastes—F80-1.0, F80-1.2, and F80-1.4, alkali-activated fly ash slag mortar was prepared by adding fine aggregates, GF, and PPF. The mix proportions of the mortars are shown in Table 4. The amount of GF and PPF was calculated based on the volume fraction of the precursor, and the water cement ratio of the mortar was set to 0.42.

## 3. Specimen Preparation and Curing

Place fly ash, slag, fine aggregate, and fiber into the mixing pot of a JJ-5 planetary sand mixer (compliant with ISO standards) for preliminary mixing. Then pour the pre-prepared activator solution into the mixing pot and stir until evenly mixed. Inject the freshly mixed pastes into a detachable steel membrane with dimensions of 40 mm × 40 mm × 160 mm and then compact it with a mortar vibration table. Cover the surface of the sample with plastic film to prevent moisture evaporation and cure at room temperature in the laboratory for 24 h. After detachment, place it in a standard curing room for curing until the specified age.

## 4. Test Procedures

### 4.1. Test Procedure for Setting Time

According to the standard GB/T 1346-2011 [57], the initial and final setting time of alkali-activated fly ash slag pastes was measured using a cement consistency setting tester.

### 4.2. Fluidity Test

According to the standard GB/T 2419-2005 [58], the fluidity of mortar was tested using a cement mortar fluidity tester.

### 4.3. Measurement of Strength

The YAW300H comprehensive mechanical testing system was used to test the flexural strength and compressive strength of alkali-activated fly ash slag pastes and mortar. The maximum load of the testing system was 300kN. The testing process was carried out in accordance with the specification GB/T 17671-2021 [59].

### 4.4. Phase Analysis

Using an XRD-6100 X-ray diffractometer, phase analysis was conducted on raw materials (fly ash, slag) and alkali-activated fly ash slag pastes. After 28 days of curing, the alkali-activated fly ash slag pastes was dried in a drying oven at 65 °C for 12 h. Then, we took fresh cross-sectional blocks from inside the pastes and ground them into a 200-mesh powder using an agate mortar and placed the powder into the X-ray diffractometer workstation for testing. The test parameters were set as follows: 2θ: 5–85°, scanning speed: 8°/min, anode target material: Cu target.

### 4.5. FT-IR Analysis

The Nicolet iN10 Fourier Transform Infrared Spectrometer (FT-IR) was used to analyze the molecular structure of raw materials (fly ash, slag) and alkali-activated fly ash slag pastes. The preparation method of alkali-activated fly ash slag paste powder was the same as that in the phase analysis experiment. The sample preparation method was KBr tablet testing, and the FT-IR testing parameters were as follows: a resolution of 2 cm^−1^, a wavenumber testing range of 400–4000 cm^−1^, and scanning time total of 32.

### 4.6. SEM Observation

The SEM test procedure was conducted according to the standard ASTM C1723-2010 [60]. An Apreo S HiVac scanning electron microscope was used to observe the micromorphology of the pastes.

### 4.7. Freeze-Thaw Cycle Test

A KDR-V5 concrete rapid freeze-thaw test box was used for freeze-thaw cycle testing of the mortar. According to the standard GB/T 41060-2021 [61], the maximum temperature at the center of the specimen is (5 ± 2) °C, and the minimum freezing temperature is (−18 ± 2) °C. A freeze-thaw cycle is completed every 4 h. The specimens were weighed after every 25 freeze-thaw cycles, the mass loss was calculated, and the peeling of the mortar surface was observed and recorded.

## 5. Results and Discussion 

### 5.1. Setting Time

The setting time of cementitious materials has a significant impact on the construction of mortar or concrete products. A too short initial setting time will affect the transportation and pouring of the mixture, while an excessively long final setting time will slow down the construction progress of the project. According to GB175-2020 [62], the initial setting time of Portland cement should not be less than 45 min, and the final setting time should not exceed 390 min.

**Figure 5:**
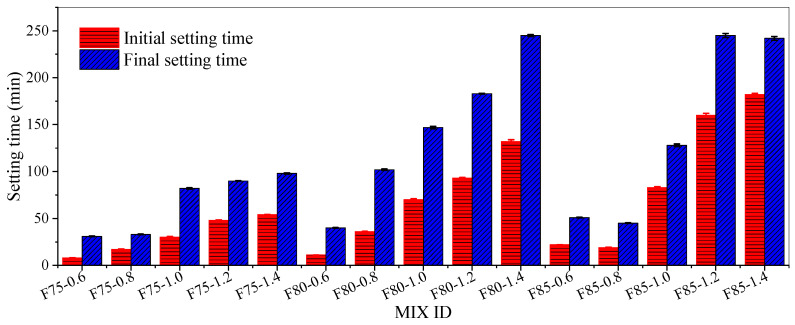
Setting time of the pastes.

Table 5 and Figure 5 show the setting time test results of alkali-activated fly ash slag pastes. The setting time of the pastes significantly increased with the proportion of fly ash in the precursor and the modulus of the alkali activator.

When the fly ash content was 75%, the setting time of the pastes was significantly lower than that of the pastes when the fly ash content was 80% and 85%. The initial setting time of the pastes in groups F75-0.6, F75-0.8, and F75-1.0 was less than 45 min. When the activator modulus was higher than 1.0, the initial setting time of the pastes was slightly higher than 45 min. However, the initial and final setting times of the F75-1.4 pastes were only 54 min and 98 min, respectively.

When the proportion of fly ash was 80%, the F80-1.4 group of pastes had the longest setting time, with initial and final setting times reaching 132 min and 245 min, respectively. When the fly ash content reached 85%, the initial and final setting times of the pastes in groups F85-0.6 and F85-0.8 were only 22 min and 51 min, respectively, and 19 min and 45 min, respectively, which were far below the requirements of GB 175-2020 standard. When the activator modulus increased to 1.4, the initial and final setting times of the pastes reached 182 min and 242 min, respectively.

At the initial stage of the reaction in the fly ash slag system activated by a mixed solution of sodium silicate and sodium hydroxide, the fly ash and slag particles underwent early dissolution, resulting in the coagulation of dissolved silicate and aluminate substances. At this stage, a large amount of elements were released, and higher alkaline cations could cause a certain degree of skeletal disorder, increase the content of non-bridging oxygen atoms, and form a small amount of weakly reactive Al-O-Al bonds. Slag contained a larger amount of Ca and Mg than fly ash, while the content of Al was lower. Due to the much weaker Ca-O-Si, Mg-O-Si, and Al-O-Al bonds compared to Al-O-Si bonds, the dissolution reaction rate of slag was faster than that of fly ash [7,63,64,65,66,67,68,69,70,71]. Therefore, as the proportion of fly ash increased, the slag content decreased, the alkali activation reaction slowed down, and the setting time was prolonged.

Due to the high content of silica in fly ash, in the alkali-activated fly ash system, the reaction began with the OH^−^ ions in the alkali activator breaking the Si-O-Si bonds [72]. As the modulus of the activator increased, the concentration of OH^−^ ions in the activator solution decreased, resulting in a weaker ability to break Si-O-Si bonds and a slower reaction process, thus prolonging the setting time.

### 5.2. Compressive Strength of Pastes

**Figure 6:**
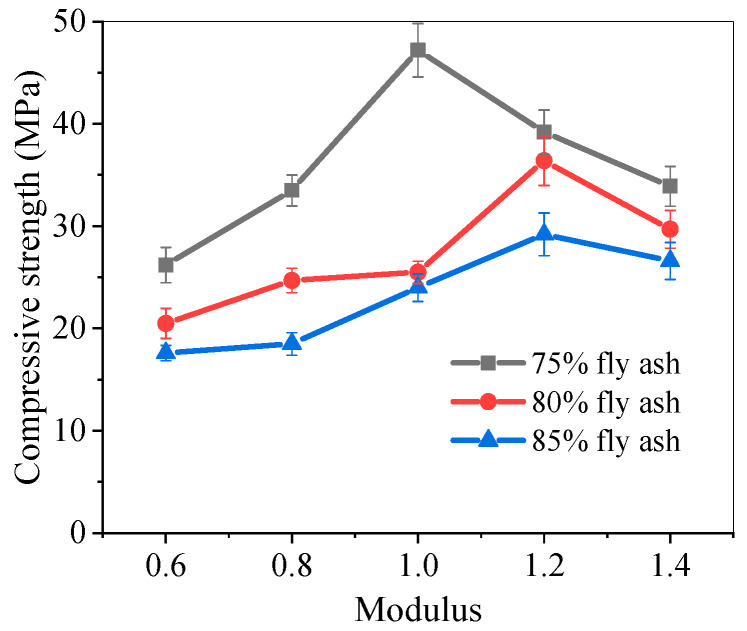
Compressive strength of alkali-activated fly ash slag pastes.

Figure 6 shows the compressive strength of alkali-activated fly ash slag pastes at 28 days. Under the same activator modulus, as the proportion of fly ash increased, the compressive strength decreased. This was mainly due to the slower reaction of fly ash compared to slag under normal temperature curing. When the proportion of fly ash was determined, the compressive strength generally increased first and then decreased with the increasing activator modulus. The compressive strength of pastes in groups F75-1.0, F80-1.2, and F85-1.2 reached the maximum compressive strength values under their respective fly ash proportions, which were 47.2 MPa, 36.4 MPa, and 29.2 MPa, respectively.

The effect of activator modulus on the compressive strength was that the development of compressive strength was affected by two factors: sodium hydroxide and sodium silicate in alkali activator. The Na^+^ in the activator could not only form sodium aluminosilicate gel, but its content could also affect the rate of gel formation. In addition, increasing the concentration of OH^−^ appropriately would significantly increase the solubility of fly ash. As the modulus of alkali activator increased, the decrease in compressive strength in the later stage was due to the excessive content of Na^+^ [73,74,75].

### 5.3. Flexural Strength of Pastes

**Figure 7:**
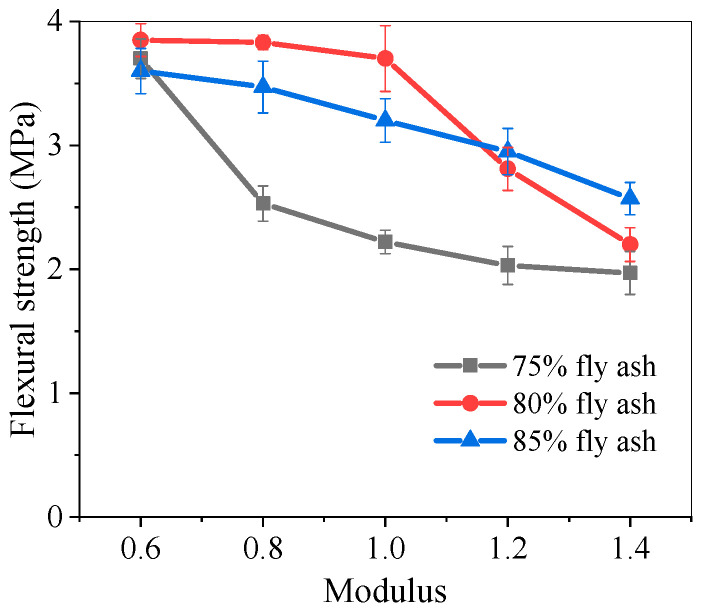
Flexural strength of alkali-activated fly ash slag pastes.

Figure 7 shows the flexural strength of alkali-activated fly ash slag pastes at 28 days. At a certain proportion of fly ash, as the activator modulus increased, the flexural strength of pastes decreased, which was significantly different from the trend of compressive strength. This may be due to the Na^+^ content in the alkaline activator exceeding the appropriate dosage. When the fly ash content was 80%, as the activator modulus increased from 0.6 to 1.0, the flexural strength of pastes slightly decreased from 3.85 MPa to 3.70 MPa. But when the activator modulus increased to 1.4, the flexural strength rapidly decreased, and the strength value of F80-1.4 was only 57.1% of F80-0.6.

The reason for the variation of flexural strength with fly ash content was that compared to alkali-activated fly ash, alkali-activated slag had a faster reaction rate, generated higher hydration heat, and caused larger cracks due to shrinkage [18]. With the increase in fly ash content, there were fewer internal structural cracks and other defects in the generated products, resulting in higher flexural strength.

### 5.4. XRD Phase Analysis

At a certain proportion of fly ash, as the activator modulus increased, some diffraction peaks in the XRD pattern of the pastes shifted, and the main components of the generated products were very complex. At obvious diffraction peaks, components such as quartz (SiO_2_) and diopside (CaMg (SiO_3_)_2_) could be identified. In areas where no obvious diffraction peaks were observed, the products included crystalline phases such as aluminosilicates (Na_6_ (AlSiO_4_)_6_, CaAl_2_SiO_6_), mullite (Al_6_Si_2_O_13_), sillimanite (Al_2_SiO_5_), siderite (FeCO_3_), and enstatite ((Mg, Fe) SiO_3_).

### 5.5. Molecular Structure Analysis

**Figure 8:**
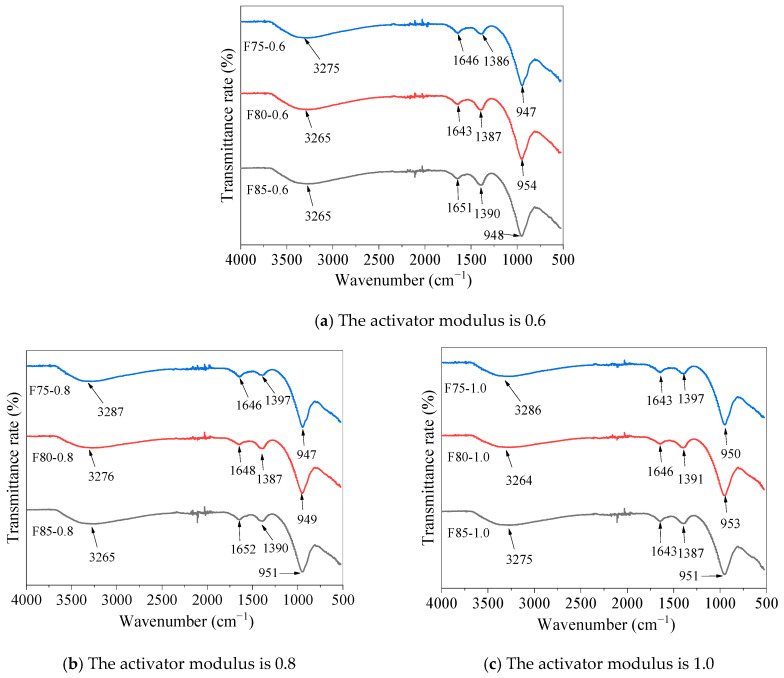

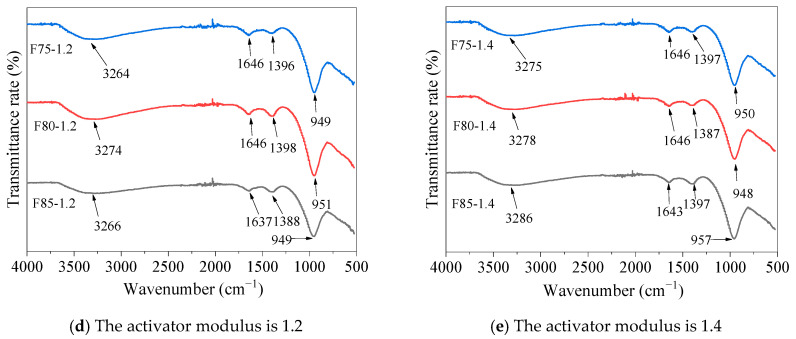
FT-IR analysis of alkali-activated fly ash slag pastes.

Figure 8 shows the FT-IR analysis of alkali-activated fly ash slag pastes. A clear absorption peak was generated at 947 to 957 cm^−1^, which was caused by the asymmetric stretching vibration of Si-O-T in C-A-S-H (where T represents tetrahedral Si or Al units) [76]. The spectral bands at 1386 to 1398 cm^−1^ were related to the O-C-O stretching vibration of carbonates [77], while the spectral bands at 1637 to 1652 cm^−1^ were caused by the H-O-H bending vibration of the molecule H_2_O [77,78,79,80]. The band at 3264 to 3287 cm^−1^ might be due to the O-H stretching of the stronger hydrogen bonding molecule H_2_O between the layers [79,80].

### 5.6. SEM Microstructure Analysis

**Figure 9:**
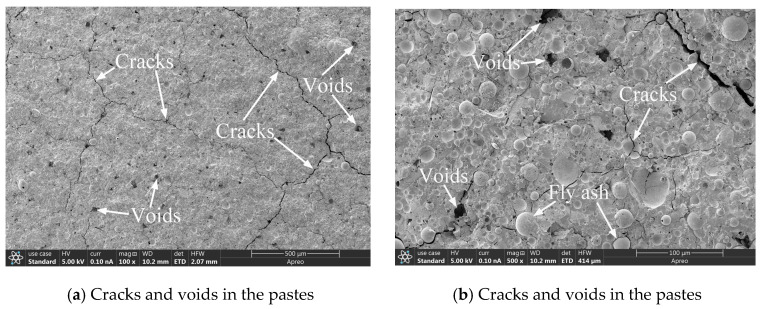

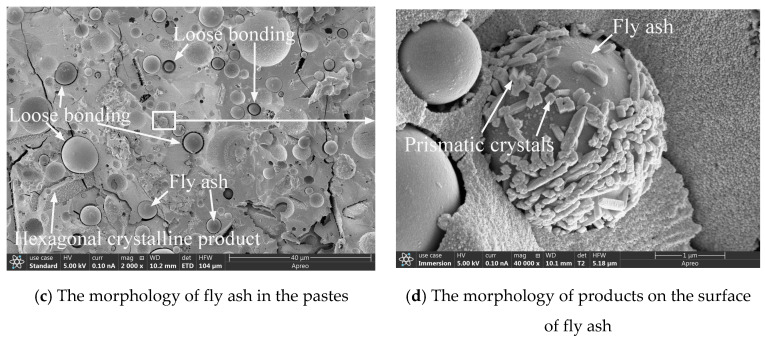
SEM images of F80-0.6.

**Figure 10:**
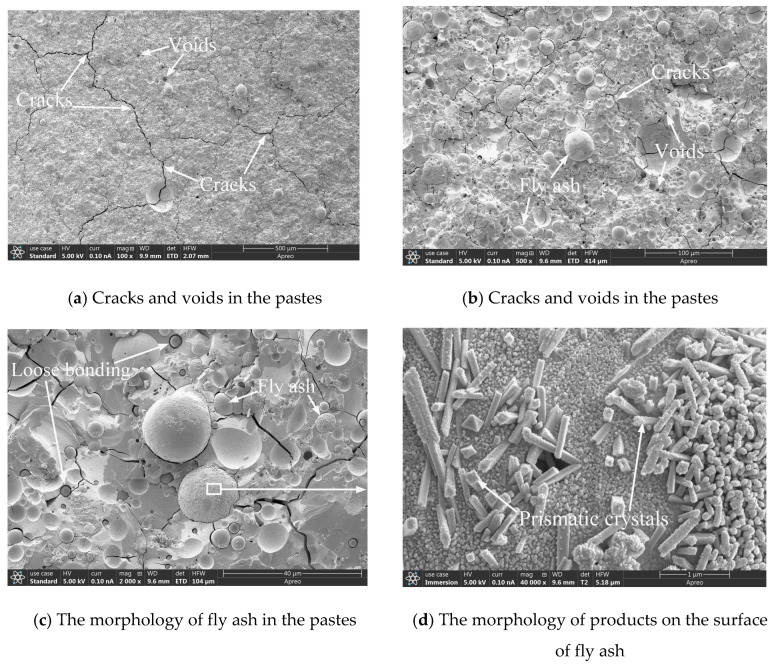
SEM images of F80-1.2.

Figure 9 and Figure 10 show SEM images of the F80-0.6 and F80-1.2 groups of pastes, respectively. In Figure 9a,b, a large number of pores and cracks could be observed in the F80-0.6 pastes when magnified 100 times and 500 times, respectively. In addition to using SEM, non-destructive testing of cracks can also be attempted using high-frequency electromagnetic techniques [81,82,83]. In Figure 10a,b, at magnifications of 100 and 500 times, cracks could also be observed in the F80-1.2 pastes, but the number of pores was much lower than that of F80-0.6. This is mainly due to the slight instantaneous setting of the newly mixed F80-0.6 composite system, which makes it difficult to compact after molding, resulting in more pores. At the same time, this is also the direct reason why the compressive strength of the F80-0.6 group of pastes is only 56.3% of the strength of the F80-1.2 group. Due to the high dosage of fly ash and curing at room temperature, fly ash particles at different reaction stages could be seen in the SEM images.

Figure 9d is an enlarged partial view of Figure 9c, showing the formation of numerous columnar crystals on the surface of fly ash particles. These columnar crystals are the same as the products generated on the surface of fly ash particles in reference [84]. Figure 10d is an enlarged partial view of the fly ash particles in Figure 10c, and it could also be seen that the surface of the fly ash particles was covered with a large number of columnar crystals. These crystals filled the gap between the fly ash particles and the surrounding matrix, making the gel structure more compact.

### 5.7. Fluidity of Mortar

**Figure 11:**
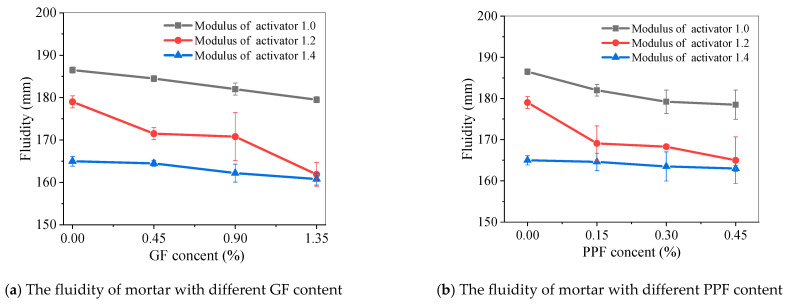
Fluidity of fiber-reinforced alkali-activated fly ash slag mortar.

Figure 11 shows the fluidity of alkali-activated fly ash slag mortar with different GF and PPF dosages. The fluidity of the mortar decreased with the increasing fiber content, and when the fiber content was determined, the fluidity of the mortar decreased with an increase in the modulus of the alkali activator.

### 5.8. Flexural Strength of Mortar

**Figure 12:**
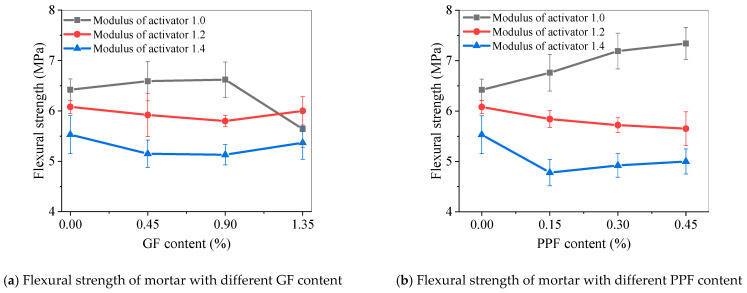
Flexural strength of fiber-reinforced alkali-activated fly ash slag mortar.

Figure 12 shows the 28-day flexural strength of alkali-activated fly ash slag mortar reinforced with GF and PPF. For GF-reinforced alkali-activated fly ash slag mortar, when the activator modulus was 1.0, the flexural strength increased first and then slightly decreased with the increasing GF content. The optimal GF content was 0.90%. At this time, the flexural strength of the M1.0-GF0.90 mortar was 6.62 MPa, which was 3.12% higher than the control M1.0-0 mortar. When the modulus of the activator increased to 1.2 and 1.4, the addition of GF slightly reduced the flexural strength.

For PPF-reinforced alkali-activated fly ash slag mortar, when the activator modulus was 1.0, the flexural strength significantly increased with the increase in PPF content. Among them, the flexural strength of M1.0-PPF0.45 was 7.34 MPa, which was 14.33% higher than that of the M1.0-0 mortar. When the modulus of the activator was 1.2 and 1.4, the addition of PPF generally led to a deterioration in the strength of the mortar.

### 5.9. Compressive Strength of Mortar

**Figure 13:**
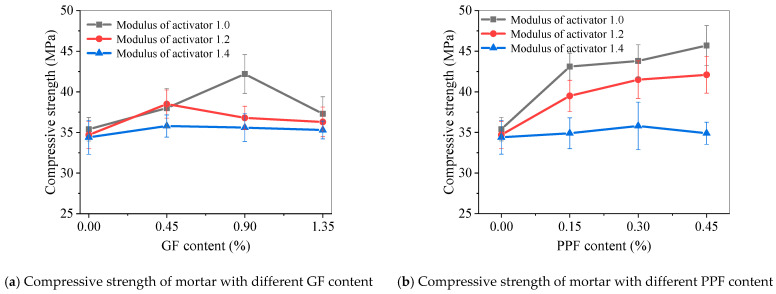
Compressive strength of fiber-reinforced alkali-activated fly ash slag mortar.

Figure 13 shows the 28-day compressive strength of GF- and PPF-reinforced alkali-activated fly ash slag mortar. The addition of GF improved the compressive strength of the mortar. When the activator modulus was 1.0, the compressive strength increased first and then slightly decreased with the increasing GF content. The optimal GF content was 0.90%. At this time, the compressive strength of M1.0-GF0.90 was 42.2 MPa, which was 19.21% higher than M1.0-0’s 35.4 MPa. When the activator modulus increased to 1.2 and 1.4, the compressive strength increased first and then decreased with the increase in GF content; 0.45% was the optimal GF dosage, and the strength values of M1.2-GF0.45 and M1.4-GF0.45 were 38.5 MPa and 35.8 MPa, respectively. Compared with the mortar without fiber, it had increased by 10.95% and 4.07%, respectively.

The addition of PPF significantly improved the compressive strength of the mortar. When the modulus of the activator was 1.0 and 1.2, the compressive strength significantly increased with the increasing PPF content. When the same amount of PPF was added, the compressive strength of the mortar with an activator modulus of 1.0 was better than that with a modulus of 1.2. The compressive strength of the M1.0-PPF0.45 mortar reached 45.7 MPa, an increase of 29.1% compared to the M1.0-0 group (i.e. the control group with 0% PPF content). When the modulus of the activator was 1.4, the improvement effect of adding PPF on the compressive strength of the mortar was relatively not significant.

### 5.10. Frost Resistance of Mortar

**Figure 14:**
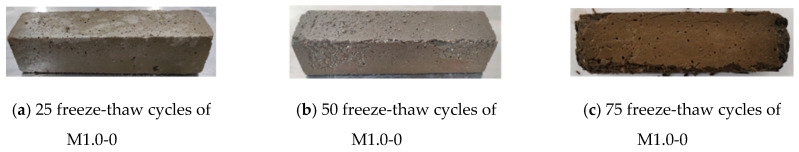

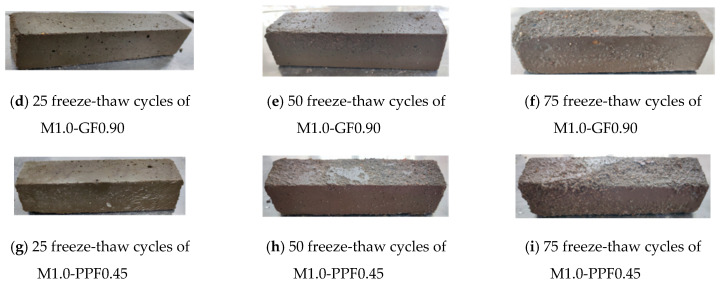
Surface morphology of freeze-thaw failure of mortar with alkali activator modulus of 1.0.

Figure 14 shows the freeze-thaw peeling of the surface of representative mortars when the activator modulus was 1.0. After 25 freeze-thaw cycles, the surface of mortars M1.0-0, M1.0-GF0.90, and M1.0-PPF0.45 remained intact. After 50 freeze-thaw cycles, the cementitious matrix on the surface of the mortars began to peel off to varying degrees, and a small amount of PPF was exposed on the surface of the M1.0-PPF0.45 mortar. After 75 freeze-thaw cycles, the detachment of mortar at the edges and corners of M1.0-0 significantly intensified. In contrast, due to the reinforcement effect of fibers, the surface of the M1.0-GF0.90 and M1.0-PPF0.45 were more intact, indicating that both GF and PPF played a significant role in improving the frost resistance of mortar.

**Figure 15:**
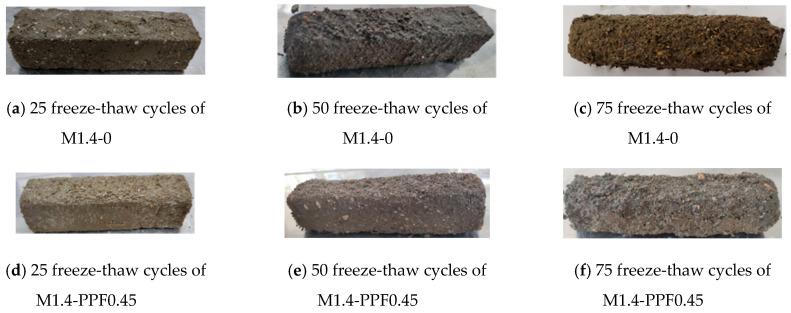
Surface morphology of freeze-thaw failure of mortar with alkali activator modulus of 1.4.

Figure 15 shows the surface morphology of freeze-thaw failure of mortar with alkali an activator modulus of 1.4. After 25 freeze-thaw cycles, the surface matrix of M1.4-0 and M1.4-PPF0.45 had undergone significant peeling. After 75 freeze-thaw cycles, all the edges and corners of the specimens had peeled off, and their shapes had become approximately cylindrical. This indicates that as the activator modulus increased to 1.4, the frost resistance of the mortar significantly decreased.

**Figure 16:**
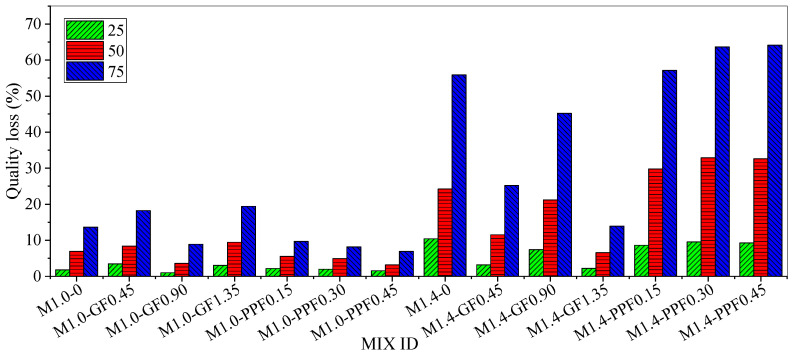
Quality loss of mortar after freeze-thaw cycles.

The quality loss of fiber-reinforced alkali-activated fly ash slag mortar after different freeze-thaw cycles is shown in Table 6 and Figure 16. When the modulus of alkali activator was 1.0, only the quality loss of the M1.0-GF0.90 mortar group was reduced after adding GF. After 75 freeze-thaw cycles, its quality loss decreased by 34.78%. The addition of PPF reduced the quality loss of the mortar. When the PPF content reached its maximum, the quality loss of M1.0-PPF0.45 was reduced by 49.30% compared to M1.0-0, indicating that the frost resistance of the mortar had been effectively improved.

When the activator modulus increased to 1.4, the quality loss of M1.4-0 after freeze-thaw cycles increased sharply compared to M1.0-0. This indicated that under freeze-thaw degradation, the frost resistance of alkali-activated fly ash slag mortar was highly sensitive to the increase in its activator modulus. When the activator modulus increased from 1.0 to 1.4, the frost resistance of the mortar with added fibers also underwent significant changes. The addition of GF reduced the loss quality of the mortar, while the addition of PPF increased the quality loss of the mortar with the increase in the freeze-thaw cycle.

## 6. Conclusions

This study systematically investigated the performance of alkali-activated fly ash slag pastes with fly ash proportions of 75% to 85% under normal temperature curing, including measures of setting time, strength, phase analysis, molecular structure analysis, and microstructures. The influence of fly ash proportion and alkali activator modulus on the above performance was analyzed. On this basis, under normal temperature curing conditions, fiber-reinforced alkali-activated fly ash slag mortars were designed and prepared. The workability, mechanical properties, and frost resistance of the mortars were studied, and the influence of the activator modulus and fiber content on the above properties of the mortar were analyzed. The following main conclusions were drawn:

(1) The setting time of alkali-activated fly ash slag pastes increased with the increasing fly ash proportion. At a specific fly ash dosage, the setting time increased with an increase in the activator modulus. The initial setting time range was from 8 to 182 min, and the final setting time was between 31 and 245 min.

(2) The compressive strength characteristics of the pastes show that they decreased with an increase in fly ash proportion at the same activator modulus, while they first increased and then decreased with an increase in the activator modulus at the same fly ash proportion. The F75-1.0 pastes achieved the highest compressive strength at 28 days, with a value of 47.2 MPa. The flexural strength decreased to varying degrees with an increase in the activator modulus, among which the F80-0.6 pastes had the highest flexural strength, which was 3.85 MPa.

(3) After adding GF and PPF, the fluidity of the mortar decreased, and the mortar with an activator modulus of 1.0 showed the most significant improvement in strength. After adding PPF, the 28-day flexural strength and compressive strength of the M1.0-PPF0.45 mortar were 7.34 MPa and 45.7 MPa, respectively, which increased by 14.33% and 29.1% compared to M1.0-0. After adding GF, the flexural and compressive strength of the M1.0-GF0.90 were 6.62 MPa and 42.2 MPa, respectively, which increased by 3.12% and 19.21% compared to M1.0-0.

(4) The frost resistance of the mortar with an activator modulus of 1.0 was significantly better than that of the mortar with an activator modulus of 1.4. Adding fibers could reduce the quality loss and surface morphology deterioration of the mortar after freeze-thaw cycles. Among them, the quality loss of M1.0-PPF0.45 mortar decreased by 49.30% compared to M1.0-0, effectively delaying the deterioration of freeze-thaw performance.

## Figures and Tables

**Table 1:** Chemical components of fly ash and slag.

Chemical Components (%)	Fly Ash	Slag
SiO_2_	58.23	29.51
Al_2_O_3_	19.21	15.32
Fe_2_O_3_	8.2	0.85
CaO	7.12	40.63
K_2_O	2.29	0.46
MgO	1.46	7.82
Na_2_O	1.32	0.41
TiO_2_	0.78	1.4
SO_3_	0.54	2.74
P_2_O_5_	0.25	-
MnO	-	0.53
Other Components	0.6	0.33

**Table 2:** Basic parameters of sodium silicate.

Testing Items	Baume Degree (20 °C)	Iron (Fe) Content	Content of Insoluble Substances in Water	Density (20 °C)	Content of Na_2_O	Content of SiO_2_	Modulus
Testing results	50 Be	0.01%	0.06%	1.53g/cm^3^	13.73%	32.35%	2.43

**Table 3:** Mix proportions of alkali-activated fly ash slag pastes.

MIX ID	Liquid/Binder Ratio	Fly (kg/m^3^)	Slag (kg/m^3^)	NaOH (kg/m^3^)	Sodium Silicate (kg/m^3^)	Water (kg/m^3^)
F75-0.6	0.52	865.4	288.5	99.5	198.2	297.0
F75-0.8	0.53	865.4	288.5	85.9	273.7	256.3
F75-1.0	0.55	865.4	288.5	75.5	331.0	225.4
F75-1.2	0.56	865.4	288.5	67.4	376.0	201.1
F75-1.4	0.57	865.4	288.5	55.5	442.0	165.5
F80-0.6	0.52	923.1	230.8	99.5	198.2	297.0
F80-0.8	0.53	923.1	230.8	85.9	273.7	256.3
F80-1.0	0.55	923.1	230.8	75.5	331.0	225.4
F80-1.2	0.56	923.1	230.8	67.4	376.0	201.1
F80-1.4	0.57	923.1	230.8	55.5	442.0	165.5
F85-0.6	0.52	980.8	173.1	99.5	198.2	297.0
F85-0.8	0.53	980.8	173.1	85.9	273.7	256.3
F85-1.0	0.55	980.8	173.1	75.5	331.0	225.4
F85-1.2	0.56	980.8	173.1	67.4	376.0	201.1
F85-1.4	0.57	980.8	173.1	55.5	442.0	165.5

**Table 4:** Mix proportions of mortar.

MIX ID	Fly (kg/m^3^)	Slag (kg/m^3^)	Sand (kg/m^3^)	NaOH (kg/m^3^)	Sodium Silicate (kg/m^3^)	Water (kg/m^3^)	GF (%)	PPF (%)
M1.0-0	360	90	1350	33.7	147.5	100.5	-	-
M1.0-GF0.45	360	90	1350	33.7	147.5	100.5	0.45	-
M1.0-GF0.90	360	90	1350	33.7	147.5	100.5	0.90	-
M1.0-GF1.35	360	90	1350	33.7	147.5	100.5	1.35	-
M1.0-PPF0.15	360	90	1350	33.7	147.5	100.5	-	0.15
M1.0-PPF0.30	360	90	1350	33.7	147.5	100.5	-	0.30
M1.0-PPF0.45	360	90	1350	33.7	147.5	100.5	-	0.45
M1.2-0	360	90	1350	30	167.6	89.6	-	-
M1.2-GF0.45	360	90	1350	30	167.6	89.6	0.45	-
M1.2-GF0.90	360	90	1350	30	167.6	89.6	0.90	-
M1.2-GF1.35	360	90	1350	30	167.6	89.6	1.35	-
M1.2-PPF0.15	360	90	1350	30	167.6	89.6	-	0.15
M1.2-PPF0.30	360	90	1350	30	167.6	89.6	-	0.30
M1.2-PPF0.45	360	90	1350	30	167.6	89.6	-	0.45
M1.4-0	360	90	1350	24.7	197	73.8	-	-
M1.4-GF0.45	360	90	1350	24.7	197	73.8	0.45	-
M1.4-GF0.90	360	90	1350	24.7	197	73.8	0.90	-
M1.4-GF1.35	360	90	1350	24.7	197	73.8	1.35	-
M1.4-PPF0.15	360	90	1350	24.7	197	73.8	-	0.15
M1.4-PPF0.30	360	90	1350	24.7	197	73.8	-	0.30
M1.4-PPF0.45	360	90	1350	24.7	197	73.8	-	0.45

**Table 5:** Setting time of alkali-activated fly ash slag pastes.

MIX ID	Initial Setting Time (min)	Final Setting Time (min)
F75-0.6	8	31
F75-0.8	17	33
F75-1.0	30	82
F75-1.2	48	90
F75-1.4	54	98
F80-0.6	11	40
F80-0.8	36	102
F80-1.0	70	147
F80-1.2	93	183
F80-1.4	132	245
F85-0.6	22	51
F85-0.8	19	45
F85-1.0	83	128
F85-1.2	160	245
F85-1.4	182	242

**Table 6:** Quality loss of mortar during freeze-thaw cycle (%).

MIX ID	25 Freeze-Thaw Cycles	50 Freeze-Thaw Cycles	75 Freeze-Thaw Cycles
M1.0-0	1.8	6.92	13.63
M1.0-GF0.45	3.46	8.39	18.26
M1.0-GF0.90	1	3.63	8.89
M1.0-GF1.35	3.04	9.47	19.43
M1.0-PPF0.15	2.19	5.56	9.72
M1.0-PPF0.30	1.97	4.92	8.16
M1.0-PPF0.45	1.54	3.2	6.91
M1.4-0	10.42	24.21	55.87
M1.4-GF0.45	3.21	11.54	25.18
M1.4-GF0.90	7.4	21.19	45.21
M1.4-GF1.35	2.22	6.6	13.92
M1.4-PPF0.15	8.64	29.78	57.11
M1.4-PPF0.30	9.57	32.89	63.66
M1.4-PPF0.45	9.26	32.65	64.14

## Data Availability

The original contributions presented in the study are included in the article, further inquiries can be directed to the corresponding author.
